# How nurses use reassurance to support the management of acute and chronic pain in children and young people: An exploratory, interpretative qualitative study

**DOI:** 10.1002/pne2.12045

**Published:** 2021-01-25

**Authors:** Bernie Carter, Jane Harris, Abbie Jordan

**Affiliations:** ^1^ Faculty of Health, Social Care and Medicine Edge Hill University Ormskirk UK; ^2^ Department of Psychology Centre for Pain Research University of Bath Bath UK

**Keywords:** children, communication, nurses, paediatric pain, parents, qualitative, reassurance

## Abstract

Reassurance in the context of pediatric pain is regarded to promote distress. Typically, spoken reassurance is reported as short, generic statements (“it's ok,” “don't worry”); little research has considered wider reassuring behaviors and actions undertaken by nurses. Most studies focus on unidirectional, dyadic relationships between reassurance and pain (parent‐to‐child, professional‐to‐child) failing to capture the inherent complexities. Adopting an exploratory, interpretative, and qualitative approach, this paper reports on findings from the qualitative interview component of a mixed‐methods study, concerning how nurses actively use reassurance when talking to children and their parents about pain. Eighteen nurses with experience of managing children's pain were recruited on completion of an international online survey (distributed by pain and children's nursing networks and via newsletter, email, and social media). All 18 nurses completed a semi‐structured interview concerning their experiences of managing children's pain working in the UK (n = 14), Canada (n = 3), and Australia (n = 1) in primary, secondary, and tertiary settings with nursing experience ranging from pre‐qualification to >20 years. Thematic analysis generated three themes which reflect the main ways in which nurses focus their reassurance within encounters with children and their parent(s): (a) on child and parent(s), (b) on the child, and (c) on the parent. Nurses generated reassurance using language, gesture, relationship building, individualizing approaches, education, and preparation. The study highlights the diversity of reassurance provided by nurses in relation to children's pain. Our study finds that when nurses reassure children about pain, they focus their reassurance in three distinct directions (child, parents, and children and parents in partnership); this has not been specifically acknowledged by previous research. We highlight the wide range of implicit and explicit reassurance actions undertaken by nurses and propose that reassurance that extends beyond limited vocalizations is part of a complex package of care that can support children's current and future pain experiences.

## 1 INTRODUCTION

Reassurance in the context of pediatric pain was precisely defined as a core code in the Child‐Adult Medical Procedure Interaction Scale (CAMPIS) handbook as “procedure‐related comments that are directed toward the child with the intent of reassuring the child about his/her conditions, or the course of the procedure”.^1^, ^2^ ^, p7^ A more recent definition is “a procedure‐related comment to child with the intent of neutralizing the situation or suggesting that the environment is nonthreatening”.^3^ ^, p1123^ Reassurance is widely regarded to promote child distress during experiences of pain.^1^, ^3^, ^4^ In painful procedures, reassuring comments by adults have been shown to precede child distress^1^ and heighten child ratings of fear.^5^ In work addressing postoperative distress, reassurance has been positively correlated with distress, and it is proposed it may maintain ongoing child distress.^3^ This counterintuitive relationship between reassurance and children's pain has been explained through three mechanisms: (a) reassurance may warn a child that an adult is anxious or that something bad is about to happen, (b) reassurance may reinforce children's apprehension and thus their distress, and (c) reassurance gives a child permission to openly express their distress.^4^, ^6^, ^7^ Reassurance accounts for more than one quarter of spontaneous adult vocalizations (parent, medical staff) made to children during medical procedures.^4^, ^8^ Additionally, experimental studies identified that parental reassurance accounted for 34%‐53% of the variance in child distress during children's painful procedures.^9^, ^10^, ^11^

However, gaps remain in the existing evidence regarding reassurance for children's pain. The majority of studies focus on parental reassurance^11^, ^12^, ^13^, ^14^ with fewer studies focusing on how nurses may use reassuring talk.^15^, ^16^ A small number of studies have examined reassurance in terms of specialisms such as oncology^8^ and physical therapy^17^ or setting such as the postanesthesia care unit.^3^ Additionally, the focus has typically involved the study of acute pain in the context of painful procedures, such as that associated with lumbar puncture,^1^ venipuncture^5^ with few studies addressing the study of reassurance in the context of chronic pain.^4^

The most common examples of spoken reassurance in the pediatric pain literature include short, generic statements such as “it's ok” and “don't worry”.^3^, ^11^, ^18^ Importantly, the different qualities of the construct of reassurance have not yet been sufficiently explored in the pediatric literature.^11^ In particular, little research has considered the range of behaviors and actions beyond these generic statements which are undertaken by nurses with the intention of reassuring children experiencing pain. Finally, the literature tends to focus on unidirectional, dyadic relationships between reassurance and pain; either reassurance of a child by their parent or by a healthcare professional. As other authors have noted, many studies fail to capture the complexities of what aspects of reassurance prompts child distress.^3^

This paper reports on findings concerning how nurses use reassurance when talking to children and their families about pain. These findings were generated from a subsidiary analysis of qualitative semi‐structured interviews that were originally undertaken as part of a mixed‐methods study which explored nurses' “pain talk” in the context of managing children's pain.

## 2 METHODS

### 2.1 Design

An exploratory, interpretative, and qualitative approach using in‐depth semi‐structured interviews.

### 2.2 Sample and recruitment

Nurses with experience of managing children's pain were recruited from an international online survey (via children's pain and nursing networks and newsletter, email, social media) that examined how nurses talk to children and their families about pain. All 141 eligible participants who completed the survey were asked whether they would be willing to participate in a follow‐up interview. Interested participants who provided their email address through the online survey received a participant information sheet from a researcher (AJ, BC) prior to providing fully informed written consent. Eligible participants were registered (or equivalent) nurses or undertaking training to obtain a formal qualification in children's nursing and aged 18 years or older. Sixty participants expressed an interest in the study, and a matrix was purposively generated to reflect the range of grades, roles, and geographic locations. The matrix was systematically worked through to follow‐up eligible participants; all who could be contacted and consented (convenience sample) within the timeframe were interviewed. No relationships existed between participants and researchers (eg, line management, close friendship). Ethics approval for the study was provided by the University of Bath and Edge Hill University.

### 2.3 Data collection

Data were collected (May 2018‐July 2018) using single, in‐depth, semi‐structured telephone, FaceTime, or Skype interviews by AJ (female, PhD, senior lecturer psychology) or BC (female, PhD, professor children's nursing). Interviews were conducted in a private room within the researcher's/participant's work or home setting, as appropriate; no other people were present during the interviews.

**FIGURE 1:**
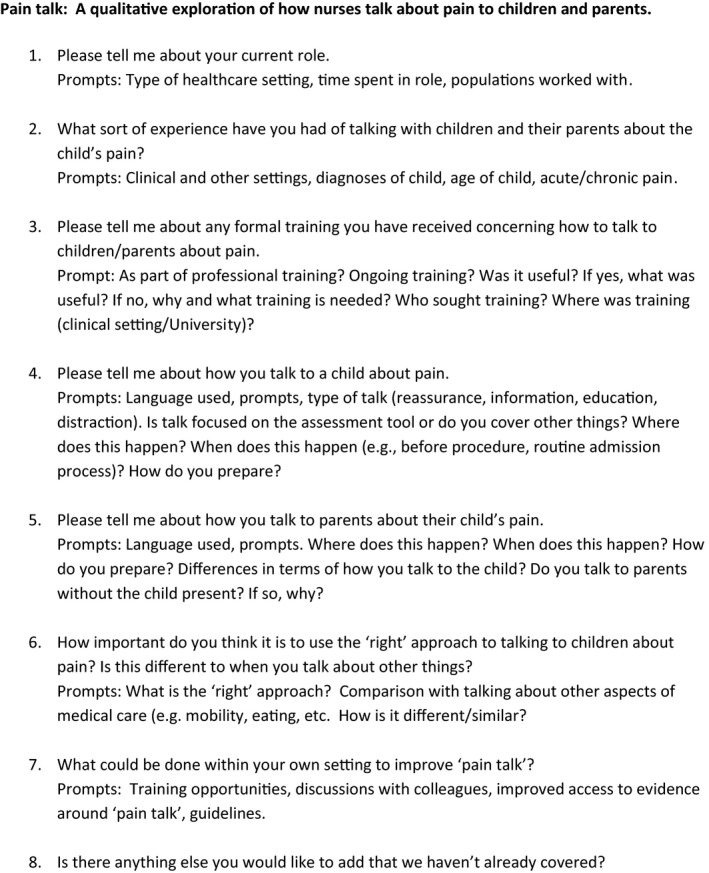
Interview topic guide

A topic guide was developed from the literature and from the authors' expertise in the fields of pediatric pain and nursing and informed by discussions with nurses working with children who experience pain. The guide was minimally refined after the first interviews had been conducted to include questions about which aspects of “pain talk” they thought were important and why. The topic guide included open‐ended questions that covered roles and pain‐related training and experience of talking to children/parents about child pain (eg, type of language, where conversations take place, types of talk). Prompts were used as appropriate (see Figure 1 for full interview guide). Although we did not share our topic guide with the participants prior to their interview, the information sheets gave a broad indication of the topic areas such as choice of language when talking about pain and challenges faced when talking to children and/or parents about pain.

Participants were not involved in the design, recruitment, or conduct of the study. Participants will be informed via email about the publication of results.

Field notes were made during/after interviews. Interviews were audio recorded and alongside informed written consent, consent was confirmed verbally at the beginning and end of each interview. Although data saturation is often considered a regulatory ideal, the most recent guidance for thematic analysis suggests that it is not a useful concept as judgements are situated and subjective.^19^

### 2.4 Data analysis

Interviews (mean 31 minutes, range 22‐55 minutes) were transcribed verbatim and analyzed in NVivo version 12 using inductive reflexive thematic analysis. Braun and Clarke's stages of familiarization, coding, producing, reviewing, and labeling themes, were followed iteratively from descriptive to interpretative analysis.^20^, ^21^ Analyses were conducted by JH (female, PhD, research fellow public health) and themes reviewed by AJ and BC. Authors actively discussed the analyses and feedback at all stages of theme development, and subsequently all authors participated in such discussions throughout theme finalization and manuscript write up. Such processes were considered critical with regard to establishing analytical quality and in particular, to ensure that author interpretations and inputs into theme development were credible and grounded in the data.

## 3 RESULTS

Eighteen nurses with experience of managing children's pain participated. Participants were working in the UK (n = 14), Canada (n = 3), and Australia (n = 1) in a range of primary, secondary, and tertiary settings with nursing experience ranging from pre‐qualification to over 20 years (Table 1).

**TABLE 1:** Summary of participants

Participant code	Job role	Setting	Country	Acute and/or chronic pain	Years since qualification
P1	Nurse Practitioner	Community	Canada	Acute	>20 y
P2	Nurse Practitioner	Community	UK	Acute & Chronic	>20 y
P3	Practice Development Nurse	Community	UK	Acute & Chronic	16‐20 y
P4	Sister	Community	UK	Acute & Chronic	5‐10 y
P5	Nurse Practitioner	Secondary	UK	Chronic	>20 y
P6	Clinical Nurse Educator	Secondary	UK	Acute & Chronic	11‐15 y
P7	Nurse Specialist	Secondary	UK	Chronic	>20 y
P8	Nurse Specialist	Secondary	UK	Chronic	>20 y
P9	Nurse Specialist	Secondary	Canada	Acute	>20 y
P10	Staff Nurse	Secondary	UK	Chronic	<5 y
P11	Senior Sister	Secondary	UK	Acute	16‐20 y
P12	Sister	Secondary	UK	Acute & Chronic	>20 y
P13	Nurse Specialist	Secondary	UK	Acute	>20 y
P14	Staff Nurse	Secondary	Canada	Acute	11‐15 y
P15	Professor	Education	Australia	Acute & Chronic	>20 y
P16	Pre‐registration Nursing Student	Education	UK	Acute	Not applicable
P17	Pre‐registration Nursing Student	Education	UK	Acute	Not applicable
P18	Pre‐registration Nursing Student	Education	UK	Acute	Not applicable

**FIGURE 2:**
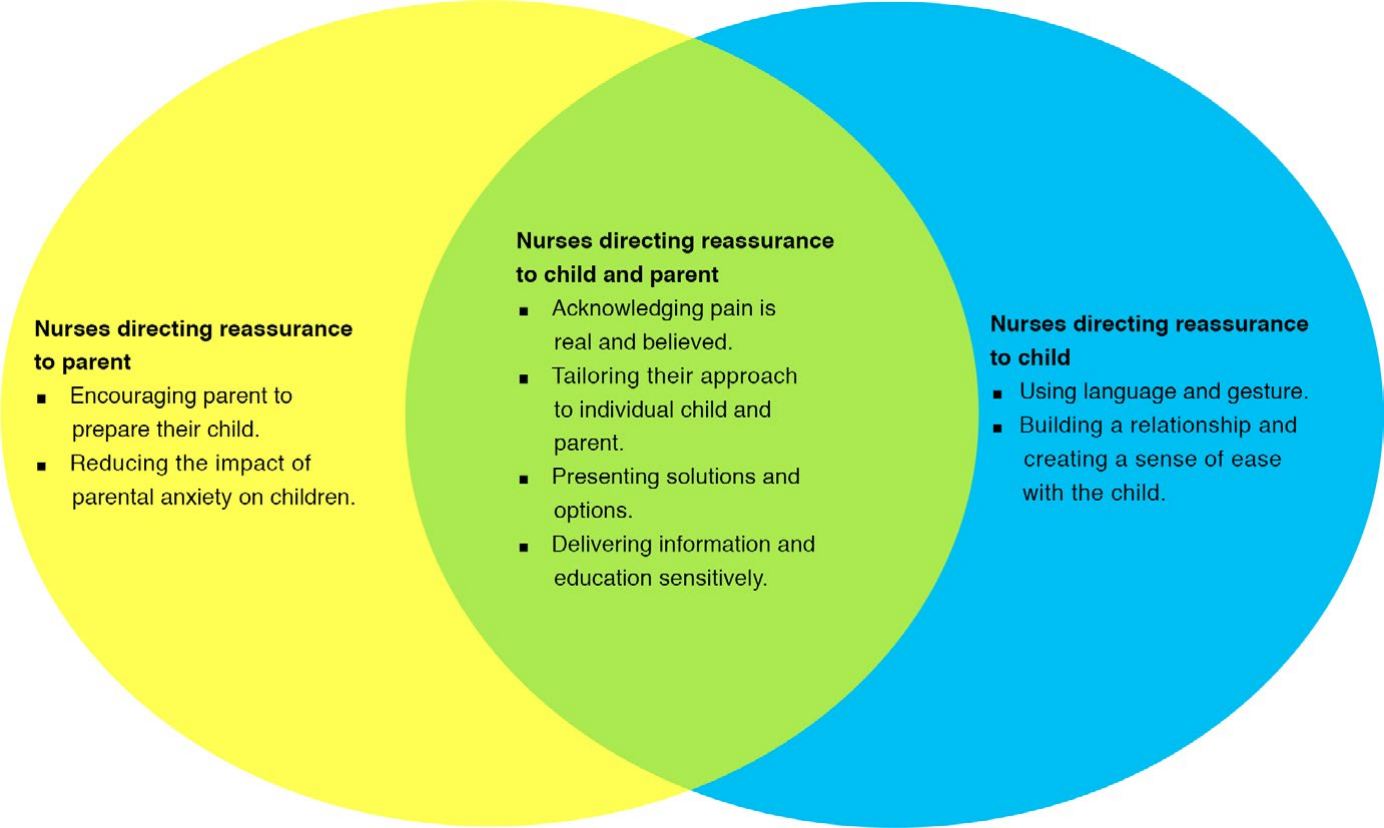
Directions and types of reassurance used by nurses for children's pain

Three main themes were generated reflecting the key ways in which nurses focus their reassurance within encounters with children and their parent: (a) focus on both the child and parent(s), (b) focus on the child, and (c) focus on the parent. Each of these themes has sub‐themes. Nurses generated reassurance in different ways including language, gesture, relationship building, individualizing approaches, education, and preparation. Our findings are presented as a framework (Figure 2); each aspect is described below with illustrative quotations from the participants. Labels are applied to quotations as follows; P (participant number), and caseload is represented as A = Acute, C = Chronic, A/C = Acute and Chronic.

### 3.1 Being a calm and knowledgeable presence: nurses providing reassurance to the child and their parents

Reassurance directed contemporaneously to both the child and parents was achieved in four main ways: acknowledging pain is real/believed; tailoring their approach to the individual child; presenting solutions and options; and using education or information.

#### 3.1.1 Acknowledging pain is real/believed

Several participants discussed both acute and chronic pain situations, highlighting the importance of communicating belief in a child's pain for the purpose of reassuring children and parents. Participants described how some parents and children talked about previous experiences of being disbelieved and had been seen by multiple healthcare professionals when seeking assistance for the child's pain, for example, “they've seen up to nine healthcare providers about their pain… they've been disbelieved” (P5‐C). Acknowledging belief in the reality of the child's pain was considered strategic when meeting parents. This aimed to alleviate potential tension as, occasionally, encounters could be confrontational due to families' previous negative experiences of being disbelieved:

…they [families] might have been round the houses a little bit, different specialities… issues with people not believing they are in pain…So you have to ease them in, they become quite tense, and sometimes ready for a bit of a fight. (P2‐A/C)

In addition to setting the scene for a positive clinical interaction, reassurance by positive reinforcement and creating a context of belief aimed to help parents and children to frame the pain in terms of what was normal and expected. Positive reinforcement and affirmation postoperatively was especially important with anxious children and parents, typically participants spoke about “how well they're doing, how it's normal to have [pain]” (P8‐C).

#### 3.1.2 Tailoring their approach to individual child and parent

Participants described tailoring their approach to providing reassurance through discussing pain in an individual way as a result of the unique needs of children. Initial conversations that helped create a sense of ease were considered important. Reassurance involved considering a range of factors such as “work[ing] at the developmental level of the child” (P5‐C) and “gauging [parents'] health literacy” (P6‐A/C). This individualized approach appeared to link with a sense of containment, aiming to reduce the shock of pain. Toys such as “a teddy with…an IV line” (P10‐C) were key to preparing children for procedures and also aimed to help desensitize children.

#### 3.1.3 Presenting solutions and options

An important way that nurses provided reassurance involved them presenting children and parents with an array of options to manage their pain. This aimed to demonstrate their commitment and motivation to work with the child and family and to reassure the child by presenting a solution for example, “okay, this has to be done, but you are going to do something about it to help.” (P8‐C). Participants acknowledged the importance of reassurance that things could be changed, typically noting that “if it isn't working then we can change things…add stuff in…there's a lot more we can do to make things better for you” (P13‐A). Vocalizing these different options and acknowledging that there could be a degree of trial and error in identifying the best pain management options was seen as an important way of building trust. Reassurance was linked to providing/creating hope by explaining:

…there are lots of options available, so if this one thing doesn't work that isn't it, we just try something else…a lot of it is trying to give them hope that things are going to get better. (P7‐C)

#### 3.1.4 Delivering education and information sensitively

Many participants described the importance of providing education and clear information to parents and children as a means of reassuring them about pain:

obviously if families and children ask specific things about things then that is information sharing as well, and reassurance. (P2‐A/C)

Important types of information‐giving to reassure included providing information about medication, describing what will happen during and after the procedure, recognizing pain, and responding to parents' specific queries and concerns. This reassurance aimed to support parents' confidence in caring for their child and understanding what is happening:

A lot of the children…they're scared to move and a lot of their parents are scared to move them, so it's just reassurance like…showing them how best to handle their children and to move them. (P13‐A)

Participants described how parents were often anxious about managing their child's pain particularly once their child had been discharged from hospital with two concerns being in tension. They described the tension around:

…[not wanting] their child to be in pain…[and not wanting] to have unnecessary things that they don't need. So often you're needing to do some education and reassurance around why we're giving medicines. (P3‐A)

The participants acknowledged that the reassuring effect of information provision was dependent on factors such as “gauging what they [families] need and trying to meet their needs” (P13‐A), and the timing and range of information as some “only want information that day, whereas some want to know exactly what's going to happen in the next two weeks” (P13‐A). The participants were sensitive to the need to meet the differing needs of children and parents. Information provision needs varied both between and within families, adding complexity to the participants' task of meeting everyone's needs. Sometimes tension occurred between children and their parents, in relation to their desired level of detail of information about pain management. For example, one adolescent (P8‐C) was satisfied with fairly generic reassurance, he explained that his mother was extremely anxious and required much more detailed information:

…about intravenous painkillers and why intravenous painkillers weren't good to be used long term. And she needed all the technical information…how strong the different painkillers were, how they worked, what was the risk to his liver and his kidneys. But he [adolescent] wasn't interested in any of that, he didn't want to talk about his liver or his kidneys or anything, he just wanted to know…what we were going to do. (P8‐C)

### 3.2 Creating a sense of comfort: nurses providing reassurance to the child

Reassurance directed primarily to the child was achieved in two main ways: through language and gesture, and through relationship building.

#### 3.2.1 Using language and gesture

Language was seen as powerful and as a potential ally, providing it was used carefully. Participants described the importance of using neutral and realistic language to help explain “this is what you'd feel” (P13‐A) when preparing children for the experience of anticipated pain. Although a few participants did use phrases intended to be generically reassuring such as “don't worry,” most were aware that “don't worry actually conveys worry” (P5‐C) which in turn could increase the child's experience of pain.

Being selective about language, being honest, acknowledging they would experience some pain were deemed to be important “as opposed to giving false reassurance and saying it won't hurt” (P1‐A). Explicit reassurance aimed to inform children about key elements such as:

how long it's likely to hurt or pinch or how long you're likely to be doing the procedure, what they can do in the meantime and then what are we going to do afterward. (P5‐C)

One participant (P15‐A/C) discussed the importance of informing children about pain in this way to help create a “realistic memory not the exaggerated memory,” potentially creating reassurance for the future:

I've got pictures that kids have drawn of needles that are eight inches long that went into their arms…showing them the equipment and going “this is the size”…showing them the straw…letting them do medical play…that can really help them reframe it to the more realistic. Not lying to them and saying it didn't happen. (P15‐A/C)

Using non‐verbal gestures was mentioned by a small number of participants as a means of reassuring children such as:

sitting at their level… reassurance…can be non‐verbal…it's just the touch of the shoulder and just the hands, those sorts of things, where you are smiling. (P2‐A/C)

#### 3.2.2 Building a relationship and creating a sense of ease with the child

Building a relationship and creating a sense of ease with each child was fundamental to reassurance and, although the final outcome was pain‐oriented, this relationship building encompassed more than just pain. Relationship building was achieved by being approachable, considerate, and creating a sense of comfort; typically, this aimed to “just sort of ease them in and settle them in” (P2‐A/C). Participants highlighted two aspects of relationship building, firstly, simply using nonprocedural talk to put the child at ease, especially the children who need a lot of support:

…we tend to spend a lot of time just sitting talking to them and their parents – not actually necessarily about their pain – just generally. (P2‐A/C)

Secondly, reassurance to help the child to become accustomed to the unfamiliar clinical environment by establishing a degree of predictability and clear expectations, such as:

…saying what's going to be happening in the day…, they can find that quite reassuring like if they're having physio or any other input. They know when they're having medications and timing it around…so that their painkillers are maximized before that happens, just to reassure them in that way. (P13‐Acute)

### 3.3 Engendering confidence: nurses providing reassurance to the parents

Reassurance directed primarily to the parent was achieved in two main ways; by encouraging parents to prepare their child and reducing the impact of parental anxiety.

#### 3.3.1 Encouraging the parent to prepare their child

Participants perceived reassurance being inherent in parents effectively preparing children for a painful procedure. Typically participants noted “we really like parents to tell the kids before they come into the clinic, we don't like it to be a surprise” (P1‐A). Participants working in the community and/or chronic pain practice talked of each procedure being part of a potentially lifelong relationship with the child noting, for example, “I actually won't do the vaccines if it's a surprise to them, cos it, we're building a relationship with these kids and it's lifelong” (P1‐A). In addition, participants felt that giving parents a defined role to play in preparing their child was also reassuring for parents who could otherwise feel uncertain and powerless to help.

…because they'd [child] seen teddy or doll or whatever beforehand they seemed to respond quite positively, and I think the parents found it quite helpful as well to prepare their child. They [parents]…said they found it quite helpful and a good way of communicating as well. (P10‐C)

#### 3.3.2 Reducing the impact of parental anxiety on children

Creating a reassuring emotional climate was deemed important as they knew that parental anxiety could be transferred to their child, typically commenting that “the child picks it all up from the parents” (P12‐A/C). Participants highlighted how parents focusing too much on their child's pain could serve to worsen their child's experience as:

you can sometimes have a child that is absolutely fine and has told you their pain score…their pain and they're having a drink or having something to eat and a chat with you and the parent will come in and say ‘ooo is it hurting? It must be hurting. It's definitely hurting’. (P11‐A)

Causes of parental anxiety included guilt that they were to blame for their child's pain and when reassurance was provided the participants ‘absolved’ parents:

these kids would come in with broken arms…I would say to Mum ‘Isn't it terrible, you just turned around for a second and they fell off that shelf’ or whatever…and you could just see the relief on the parent's face. (P15‐A/C)

Managing other causes of parental anxiety such as feeling solely responsible for their child's pain management could be achieved by working with parents and picking up that responsibility. One participant noted that when “nurses will lead giving the meds…it just helps take the pressure away from the parents” (P12‐A/C). Reassurance also was engendered through generating confidence and supporting the parent to support their child as:

they need to know what's going on…have confidence in you, so they know what's happening; they can make sure their child is kept calm. (P8‐C)

Steps taken by participants to reassure and alleviate the impact of parental anxiety on children's pain included keeping the parent as involved as possible; this included “walk[ing] people through that experience and hopefully make them feel as comfortable as possible” (P15‐A/C). Reassurance and relationship building with parents, meant parents were better equipped to ensure their child is kept calm and consequently reduce the child's experience of pain.

## 4 DISCUSSION

This study highlights the diversity in the nature of reassurance provided by nurses in relation to children's pain. The study illustrates that nurses' reassurance for children's pain is directed at the child (through language and gesture, and relationship building), the parent (by encouraging parents to prepare their child and reducing the impact of parental anxiety) and both the child and parent simultaneously (believing pain, individualized approach, presenting solutions/options, and education/information as reassurance). This reveals a more complex presentation of how nurses use reassurance which moves beyond the dyadic, unidirectional approach of either (a) parents reassuring their children or (b) nurses reassuring children reported in the existing pediatric literature.^4^

Our findings demonstrate numerous types of reassurance undertaken which move beyond the short, generic ‘empty’^22^ reassurance statements (such as “it's ok” and “don't worry”) described in the existing literature.^3^, ^11^, ^18^ There are similarities between the reassurance described by participants and the Holt and Pincus'^23^ work on how health professionals offer reassurance to adults experiencing lower back pain. Holt and Pincus^23^ identify two types of reassurance: implicit reassurance such as data gathering and relationship building with patients, and explicit reassurance which includes both generic and cognitive reassurance. Although this framework has been developed in adult populations, it is a useful comparator for this study as it reflects the complexities of nurses' practices, acknowledges that children will have varying experiences of pain and recognizes that nurses are often concurrently communicating with both children and adults about the child's pain.

We have identified that participants engaged in two types of implicit reassurance similar to those described by Holt and Pincus^23^: individualizing their approach for parents and children and relationship building with the child. Developing a contextualized, individualized understanding of the child's pain requires the nurse to gather data about the specific and often different needs and concerns of the child and parent; this exchange generates both reassurance and a more complete picture of the child's pain.^24^ Additionally, relationship building was evident in the effort that nurses made to put children at ease in unfamiliar clinical environments, an action that nurses know is important,^25^, ^26^ but that is not typically seen as reassurance.

Participants also discussed several forms of explicit cognitive reassurance. Participants emphasized the importance of neutral and realistic language and gesture^27^ with nurses demonstrating their credibility and authenticity; both important aspects of successful reassurance.^28^ This sense of credibility also provided the foundation for tailored information‐sharing practices including providing hope to parents and children by vocalizing the different options/solutions available to manage pain and creating opportunities for shared decision‐making.^29^ Other research advocates the importance of supporting children's literacy through providing clear information tailored to their needs and available in a timely manner before their procedure.^26^ Generic reassurance was also used to increase credibility. By communicating belief in the child's pain, a factor known to be important to children and parents,^24^ the nurses established a context of trust for further interactions.

Encouraging parents to prepare their child before painful procedures was another form of cognitive reassurance. Previous studies show parents are often reluctant to provide information to children about procedures due to misconceptions that this might heighten children's anxiety.^30^ The nurses recognized that they had a role to play in encouraging and empowering parents to provide information to their child. Nurses in this study also recognized the cyclical relationship between parental and child anxiety about pain. Other studies also note that a parent who looks or sounds fearful or anxious can cause a child to become more distressed which can in turn increase the parent's anxiety and fear.^4^, ^8^ With the aim of limiting this cyclical relationship, nurses in this study reported using generic reassurance practices such as keeping parents involved and calm to reduce the impact of parental anxiety on children.

The strength of this study is its use of in‐depth qualitative interviews to explore the types of reassurance nurses used when talking to children and their parents about pain in a way which had not been done by previous experimental^10^ and survey^31^, ^32^, ^33^ studies. The study recruited nurses from a range of specialisms, settings, years' experience, and international geographical locations. While these varied perspectives are a strength of the study, most were working in the UK which limits the international comparisons that can be made about reassurance practices. Also, while the 18 participants were purposefully selected, they were chosen from a self‐selecting sample of 60 nurses recruited from existing pain‐focused networks who may have different perspectives from a more generic sample of nurses.

In conclusion, our study finds that when nurses reassure children about pain, they focus their reassurance in three distinct directions, child, parents, and children and parents in partnership, which has not been specifically acknowledged by previous research. It also highlights the wide range of implicit and explicit reassurance actions undertaken by nurses. However, by highlighting these complexities, our study also reveals some gaps in the existing evidence. By focusing on what nurses say they do, our findings only describe reassurances given by nurses (adults) to children and does not acknowledge that children's response to pain may have a reassuring impact on parents and nurses particularly among older children. This study highlights that the current evidence base in this area is theoretically thin.^34^ Further research on parents and children's perspectives on reassurance and application of relevant social learning and behavior change theories are needed to more fully understand the relationships between reassurance and children's experiences of pain. So in the face of the current zeitgeist that reassuring children in pain promotes distress,^11^ our findings suggest that reassurance that extends beyond limited ‘empty’^22^ vocalizations is part of a complex package of care that can support children's current and future pain experiences.

## AUTHOR CONTRIBUTIONS

AJ and BC conceived, designed, and undertook data analysis. JH contributed to data analysis and literature searching. AJ, BC, and JH contributed to writing the paper.

## Data Availability

Data available on request due to privacy/ethical restrictions.

## References

[1] Blount RL , Corbin SM , Sturges JW , Wolfe VV , Prater JM , Denise JL . The relationship between adults' behavior and child coping and distress during BMA/LP procedures: a sequential analysis. Behav Ther. 1989;20(4):585‐601.

[2] Blount RL . The Child‐Adult Medical Procedure Interaction Scale (CAMPIS) and The Child‐Adult Medical Procedure Interaction Scale‐Revised (CAMPIS‐R); 1997:1–16. 10.1093/jpepsy/22.1.73 9019049

[3] Martin SR , Chorney JM , Cohen LL , Kain ZN . Sequential analysis of mothers' and fathers' reassurance and children's postoperative distress. J Pediatr Psychol. 2013;38(10):1121‐1129. 23962770 10.1093/jpepsy/jst061 PMC3809730

[4] McMurtry CM , McGrath PJ , Chambers CT . Reassurance can hurt: parental behavior and painful medical procedures. J Pediatr. 2006;148(4):560‐561. 16647425 10.1016/j.jpeds.2005.10.040

[5] McMurtry CM , Chambers CT , McGrath PJ , Asp E . When, “don't worry” communicates fear: children's perceptions of parental reassurance and distraction during a painful medical procedure. Pain (Amsterdam). 2010;150(1):52‐58. 10.1016/j.pain.2010.02.021 20227831

[6] Chambers CT , Craig KD , Bennett SM . The impact of maternal behavior on children's pain experiences: an experimental analysis. J Pediatr Psychol. 2002;27(3):293‐301. 11909936 10.1093/jpepsy/27.3.293

[7] Smith RW , Shah V , Goldman RD , Taddio A . Caregivers' responses to pain in their children in the emergency department. Arch Pediatr Adolesc Med. 2007;161(6):578‐582. 17548763 10.1001/archpedi.161.6.578

[8] Spagrud LJBA , von Baeyer CLP , Ali KMD , et al. Pain, distress, and adult‐child interaction during venipuncture in pediatric oncology: an examination of three types of venous access. J Pain Symptom Manage. 2008;36(2):173‐184. 18400458 10.1016/j.jpainsymman.2007.10.009

[9] Walker LS , Williams SE , Smith CA , Garber J , Van Slyke DA , Lipani TA . Parent attention versus distraction: impact on symptom complaints by children with and without chronic functional abdominal pain. Pain. 2006;122(1‐2):43‐52. 16495006 10.1016/j.pain.2005.12.020 PMC3232036

[10] Cohen LL , Rodrigues NP , Lim CS , et al. Automated parent‐training for preschooler immunization pain relief: a randomized controlled trial. J Pediatr Psychol. 2015;40(5):526‐534. 25638483 10.1093/jpepsy/jsu162 PMC4502392

[11] McMurtry CM , McGrath PJ , Asp E , Chambers CT . Parental reassurance and pediatric procedural pain: a linguistic description. J Pain. 2007;8(2):95‐101. 16949882 10.1016/j.jpain.2006.05.015

[12] Bush JP , Melamed BG , Sheras PL , Greenbaum PE . Mother‐child patterns of coping with anticipatory medical stress. Health Psychol. 1986;5(2):137‐157. 3732228 10.1037//0278-6133.5.2.137

[13] Gonzalez JC , Routh DK , Armstrong FD . Effects of maternal distraction versus reassurance on children's reactions to injections. J Pediatr Psychol. 1993;18(5):593‐604. 8295082 10.1093/jpepsy/18.5.593

[14] Manimala MR , Blount RL , Cohen LL . The effects of parental reassurance versus distraction on child distress and coping during immunizations. Children's Health Care. 2000;29(3):161‐177.

[15] Sweet SD , McGrath PJ . Relative importance of mothers' versus medical staffs' behavior in the prediction of infant immunization pain behavior. J Pediatr Psychol. 1998;23(4):249‐256. 9718898 10.1093/jpepsy/23.4.249

[16] Cohen LL , Bernard RS , Greco LA , McClellan CB . A child‐focused intervention for coping with procedural pain: are parent and nurse coaches necessary? J Pediatr Psychol. 2002;27(8):749‐757. 12403865 10.1093/jpepsy/27.8.749

[17] Miller AC , Johann‐Murphy M , Zhelezniak V . Impact of the therapist‐child dyad on children's pain and coping during medical procedures. Dev Med Child Neurol. 2001;43(2):118‐123. 11221899 10.1017/s0012162201000202

[18] Jenkins BN , Granger DA , Roemer RJ , Martinez A , Torres TK , Fortier MA . Emotion regulation and positive affect in the context of salivary alpha‐amylase response to pain in children with cancer. Pediatr Blood Cancer. 2018;65(6):e26973. 29350481 10.1002/pbc.26973 PMC6746182

[19] Braun V , Clarke V . To saturate or not to saturate? Questioning data saturation as a useful concept for thematic analysis and sample‐size rationales. Qual Res Sport Exerc Health. 2019;1‐16. https://www.tandfonline.com/doi/full/10.1080/2159676X.2019.1704846

[20] Braun V , Clarke V . Using thematic analysis in psychology. Qual Res Psychol. 2006;3(2):77‐101.

[21] Braun V , Clarke V . Reflecting on reflexive thematic analysis. Qual Res Sport Exerc Health. 2019;11(4):589‐597.

[22] Bray L . ‘Empty’ reassurance. In: Carter B , ed. Verbal Communication ed; 2020.

[23] Holt N , Pincus T . Developing and testing a measure of consultation‐based reassurance for people with low back pain in primary care: a cross‐sectional study. BMC Musculoskelet Disord. 2016;17:277. 27405870 10.1186/s12891-016-1144-2 PMC4941026

[24] Jordan AL , Eccleston C , Osborn M . Being a parent of the adolescent with complex chronic pain: an interpretative phenomenological analysis. Eur J Pain. 2007;11(1):49‐56. 16458550 10.1016/j.ejpain.2005.12.012

[25] Salmela M , Salanterä S , Aronen ET . Coping with hospital‐related fears: experiences of pre‐school‐aged children. J Adv Nurs. 2010;66(6):1222‐1231. 20546356 10.1111/j.1365-2648.2010.05287.x

[26] Bray L , Appleton V , Sharpe A . ‘If I knew what was going to happen, it wouldn't worry me so much’: children's, parents' and health professionals' perspectives on information for children undergoing a procedure. J Child Health Care. 2019;23(4):626‐638. 31431048 10.1177/1367493519870654

[27] Levetown M . Communicating with children and families: from everyday interactions to skill in conveying distressing information. Pediatrics. 2008;121(5):e1441‐60. 18450887 10.1542/peds.2008-0565

[28] Espezel HJE , Canam CJ . Parent–nurse interactions: care of hospitalized children. J Adv Nurs. 2003;44(1):34‐41. 12956667 10.1046/j.1365-2648.2003.02765.x

[29] Carter B . Pain narratives and narrative practitioners: a way of working ’in‐relation’ with children experiencing pain. J Nurs Manag. 2004;12(3):210‐216. 15089959 10.1046/j.1365-2834.2003.00440.x

[30] Sng QW , Taylor B , Liam JL , Klainin‐Yobas P , Wang W , He H‐G . Postoperative pain management experiences among school‐aged children: a qualitative study. J Clin Nurs. 2013;22(7‐8):958‐968. 23311588 10.1111/jocn.12052

[31] Bandstra NF , Skinner L , Leblanc C , et al. The role of child life in pediatric pain management: a survey of child life specialists. J Pain. 2008;9(4):320‐329. 18201933 10.1016/j.jpain.2007.11.004

[32] Swiggum M , Hamilton ML , Gleeson P , Roddey T , Mitchell K . Pain assessment and management in children with neurologic impairment: a survey of pediatric physical therapists. Pediatr Phys Ther. 2010;22(3):330‐335. 20699786 10.1097/PEP.0b013e3181ea8d7d

[33] Chorney JM , Tan ET , Kain ZN . Adult‐child interactions in the postanesthesia care unit: behavior matters. Anesthesiology. 2013;118(4):834‐841. 23254147 10.1097/ALN.0b013e31827e501b PMC3789592

[34] Linton SJ , McCracken LM , Vlaeyen JW . Reassurance: help or hinder in the treatment of pain. Pain. 2008;134(1‐2):5‐8. 18035496 10.1016/j.pain.2007.10.002

